# Evolutionary relationships in Panicoid grasses based on plastome phylogenomics (Panicoideae; Poaceae)

**DOI:** 10.1186/s12870-016-0823-3

**Published:** 2016-06-18

**Authors:** Sean V. Burke, William P. Wysocki, Fernando O. Zuloaga, Joseph M. Craine, J. Chris Pires, Patrick P. Edger, Dustin Mayfield-Jones, Lynn G. Clark, Scot A. Kelchner, Melvin R. Duvall

**Affiliations:** Department of Biological Sciences, Northern Illinois University, 1425 W. Lincoln Hwy, DeKalb, IL 60115-2861 USA; Instituto de Botánica Darwinion, Labardén 200, Casilla de Correo 22, B1642HYD, San Isidro, Buenos Aires Argentina; Jonah Ventures, Manhattan, KS 66502 USA; Ecology, Evolution and Organismal Biology, 251 Bessey Hall, Iowa State University, Ames, IA 50011-1020 USA; Biological Sciences, Idaho State University, 921 S. 8th Ave, Pocatello, ID 83209-8007 USA; Biological Sciences, University of Missouri, 371b Bond Life Sciences Center, Columbia, MO 65211 USA; Department of Horticulture, Michigan State University, East Lansing, MI 48823 USA; Donald Danforth Plant Science Center, 975 North Warson Rd, St. Louis, MO 63132 USA

**Keywords:** Grasses, mtDNA, Next generation sequencing, Panicoideae, *Paspalum*, Phylogenomics, Plastome, Poaceae, ptDNA, Subtribal systematics

## Abstract

**Background:**

Panicoideae are the second largest subfamily in Poaceae (grass family), with 212 genera and approximately 3316 species. Previous studies have begun to reveal relationships within the subfamily, but largely lack resolution and/or robust support for certain tribal and subtribal groups. This study aims to resolve these relationships, as well as characterize a putative mitochondrial insert in one linage.

**Results:**

35 newly sequenced Panicoideae plastomes were combined in a phylogenomic study with 37 other species: 15 Panicoideae and 22 from outgroups. A robust Panicoideae topology largely congruent with previous studies was obtained, but with some incongruences with previously reported subtribal relationships. A mitochondrial DNA (mtDNA) to plastid DNA (ptDNA) transfer was discovered in the *Paspalum* lineage.

**Conclusions:**

The phylogenomic analysis returned a topology that largely supports previous studies. Five previously recognized subtribes appear on the topology to be non-monophyletic. Additionally, evidence for mtDNA to ptDNA transfer was identified in both *Paspalum fimbriatum* and *P. dilatatum*, and suggests a single rare event that took place in a common progenitor. Finally, the framework from this study can guide larger whole plastome sampling to discern the relationships in Cyperochloeae, Steyermarkochloeae, Gynerieae, and other *incertae sedis* taxa that are weakly supported or unresolved.

**Electronic supplementary material:**

The online version of this article (doi:10.1186/s12870-016-0823-3) contains supplementary material, which is available to authorized users.

## Background

Panicoideae are the second largest subfamily in Poaceae (grass family), comprising over 212 genera and approximately 3316 species [1]. This large subfamily contains many plants of economic interest: lawn grasses (*Eremochloa ophiuroides*, *Paspalum notatum*, *Stenotaphrum secundatum*; [2]), biofuel stocks (*Miscanthus* × *giganteus*, [3]; *Panicum virgatum*, [4]), a source of antitumor compounds in cancer research (*Coix lacryma-jobi*, [5]), and most importantly as crops (*Zea mays* (corn), *Saccharum officinarum* (sugarcane), and *Sorghum bicolor* (sorghum); [6]). Specifically, the importance of *Z. mays* is overwhelming since it accounts for 94 % of all cereal consumption, and over 717 million metric tons are produced each year for products such as starch, sweeteners, beverages, industrial alcohol, fuel ethanol, and oil [7]. Panicoideae are also major components of C_4_ grasslands [8] such as the tall grass prairies of central North America [9]. Understanding the ecological roles of *Andropogon gerardii* (big bluestem), *Panicum virgatum* (switchgrass), *Schizachyrium scoparium* (little bluestem), *Sorghastrum nutans* (Indian grass), and other C_4_ grasses in a phylogenetic context is key to successful restoration and management of this ecosystem [10, 11]. Thus, there is great interest in better understanding the phylogenetic relationships among Panicoideae due to the economic and ecological importance of this group.

Over the past few decades, our understanding of the systematics and phylogeny of Panicoideae has advanced. As of 2001, at least seven tribes were recognized in the subfamily [6]. Significant revisions to the subfamily have occurred, with the removal of Isachneae [12], and the submergence of Centothecoideae into Panicoideae [13]. A more recent and significant change was the splitting of the Paniceae into Paniceae and Paspaleae based on chromosome numbers and molecular data. The pantropical, x = 9 species remained Paniceae, while the primarily American, x = 10 species became Paspaleae [14]. The current number of tribes in Panicoideae is twelve, with three large groups: Paniceae, Paspaleae and Andropogoneae, and nine smaller groups: Arundinelleae, Chasmanthieae, Centotheceae, Cyperochloeae, Gynerieae, Steyermarkochloeae, Thysanolaeneae, Tristachyideae, and Zeugiteae [15]. From this newest taxonomy, two phylogenetic positions have been proposed for the Panicoideae within the large PACMAD (Panicoideae, Aristidoideae, Chloridoideae, Micairoideae, Arundinoideae, Danthonioideae) clade: 1) The Aristidoideae are sister to the remaining PACMAD grasses [15, 16], and 2) a newly proposed alternate hypothesis [17] that Panicoideae are sister to the rest of the PACMAD clade.

Most of these revisions to the Panicoideae were based on analyses of morphological data as well as multi-locus molecular data. The most recent revision by Soreng et al. [15] was based on two chloroplast genome (plastome) markers, *ndhF* and *matK*. Other recent studies supplemented molecular data with structural data; Sánchez-Ken and Clark [13] used three plastome markers, one nuclear marker, and 58 structural characters and Morrone et al. [14] used one plastome marker and 57 morphological characters. All of these studies were able to produce trees that contained some phylogenetic resolution, but still lacked information that could fully define relationships. Over half of the nodes are either unresolved polytomies or have low support values with maximum likelihood or maximum parsimony bootstrap values less than 80 % or posterior probabilities less than 0.95 [13, 14]. An analysis of plastome loci was recently reported by Washburn et al. [18]. The 78 protein coding loci analyzed in that study indicated monophyly of six subtribes of Paniceae and 4–7 origins of C_4_ photosynthesis. This is the only published analysis of Panicoideae to date that can be considered to be at the scale of plastome-phylogenomics, although complete plastomes were not produced or analyzed.

The ability to sequence and analyze full plastomes has accelerated phylogenomic studies in Poaceae. The breadth of research based on complete plastomes range from answering questions about deeply diverging subfamilies of grasses [19, 20], to divergences among New World Bambusoideae congruent with biogeography [21, 22], to phylogenomic relationships within and between grass subfamilies [17, 23–26]. In previous phylogenetic studies, the use of full plastomes has yielded improved estimates of species relationships with better resolution and higher support values, compared to the use of data partitions that only include coding sequences [21, 23, 26, 27]. Due to newer sequencing technologies, entire grass plastomes can readily be generated from next generation sequencing (NGS) read pools, and this is reflected in recent phylogenomic literature [17, 19, 22–26, 28–30].

In this study, 35 newly sequenced plastomes combined with 15 published plastomes were used to estimate a robust phylogeny for Panicoideae in the broad context of 22 outgroup Poaceae. With this large data set, we addressed three specific objectives. The first was to determine whether plastome phylogenomic relationships are congruent with the tribal and subtribal taxonomic relationships determined in previous studies [14, 15]. Complete plastomes were determined and analyzed for this objective to seek higher levels of support than in previous studies. A second phylogenetic objective was to revisit two alternative hypotheses for the topology of the PACMAD clade [16, 17, 31]. This entailed somewhat more extensive sampling of outgroup Poaceae and SH testing. The third objective was to look for and describe rare genomic mutation events in the subfamily. For this objective all coding and noncoding sequences of the full plastomes were investigated for what are likely to be low frequency events.

## Methods

### Sampling

The sampling in this project included the sequencing of 35 new complete plastomes from 18 subtribes found in 10 of the 12 tribes of Panicoideae emphasizing genera that are currently underrepresented in the available data on NCBI [32]. With those NCBI sequences, the sampling in Panicoideae included 50 complete plastomes. The outgroup taxa were selected to include representatives for all available Poaceae subfamilies. For the rest of the PACMAD clade we included two Chloridoideae, one Danthonioideae, one Micrairoideae, and two Aristidoideae. For the BOP clade we chose five Pooideae, four Bambusoideae and three Oryzoideae. The earliest diverging lineages were also included to represent a complete Poaceae phylogeny. Destructive sampling from herbarium material was performed with permissions from curatorial staff. Seedlings were grown from germplasm stocks obtained from the USDA National Plant Germplasm System (http://www.ars-grin.gov/npgs/). Vouchers and USDA accession numbers are indicated (Additional file 1: Table S1).

### Extraction

DNA extractions of Panicoideae samples were performed on young, green leaf tissue, silica dried leaf tissue or herbarium specimens using the DNeasy Plant Mini kit (Qiagen, Valencia, CA, USA) according to the manufacturer's instructions after homogenization of the tissue in liquid nitrogen.

### Library preparation

Samples were prepared for NGS using two different protocols. Some DNAs were diluted to 2 ng/μl, sheared into ~300 bp fragments (Bioruptor sonication, Diagenode, Denville, New Jersey, USA), purified and concentrated. Then, the TruSeq low throughput protocol (gel method) was used following the manufacturer's protocol (Illumina, San Diego, California, USA). The remaining DNA samples were diluted to 2.5 ng/μl (50 ng total) for Nextera and 0.2 ng/μl (1 ng total) for Nextera XT. Single end libraries were prepared using the standard protocol of the Illumina Nextera or Nextera XT DNA Sample Preparation kit and a dual index adaptor was used. All libraries were then sequenced at the core DNA facility at Iowa State University (Ames, Iowa, USA) on an Illumina HiSEq 2000 instrument. Library preparation methods are summarized (Additional file 2: Table S2).

### NGS Plastome assembly and verification

Illumina reads were filtered and assembled following the methods used in Wysocki et al. [33]. The reads were filtered by removing low scoring sequences and short reads (DynamicTrim version 2.1, LengthSort version 2.1) [34], with default settings. A Velvet version 1.2.08 [35] (http://www.ebi.ac.uk/~zerbino/velvet/) iterative *de novo* assembly [33] was performed on TruSeq and Nextera data with k-mers set from 19–85 bp with intervals of six. Contigs were then combined into one file and a final assembly was performed with the previous settings. For Nextera XT data, SPAdes v.3.5.0 (http://bioinf.spbau.ru/spades) [36] was used for *de novo* assembly with k-mers as above. CD-Hit version 4.6 [37] was used to remove redundant sequences in the final contig file. Then ACRE [33] was used to scaffold contigs together.

The ACRE scaffolds and reads were imported into Geneious Pro version 6.1.8 [38] (Biomatters Ltd., Auckland, New Zealand), and contigs for each sample were aligned to a closely related plastome using the MAFFT version 7.017 [39] plugin in Geneious. Finally the gaps were closed by *in silico* genome walking. The reads were mapped to the contigs and the majority of reads with at least 30 bp overlap at the end of the contig were then concatenated to the contig, minus the overlap. A final verification of each plastome was performed by mapping reads to their respective complete assembly and mean read depth was determined [33] (Additional file 3: Table S2).

### Plastome annotation

Plastome annotations were performed in Geneious Pro version 6.1.8 using the pairwise align function. A reference plastome for each new accession was determined by choosing a closely related species that was banked at NCBI. The annotations from the previously banked plastome were transferred to the new plastome. The coding sequences were examined and position boundaries were adjusted to preserve reading frames. The endpoints of the inverted repeats (IR) were located using the methods from Burke et al. [21]. BLAST [40] was used to locate IR boundaries by comparing the assembled sequence, which had small portions of the IR regions flanking the rest of the assembly, against itself. The transition points that showed an orientation change from plus/plus to plus/minus indicated the IR boundary. Geneious Pro’s motif feature was then used to flag these boundaries for annotation.

### Phylogenomic analyses

A plastome DNA (ptDNA) matrix was assembled for 72 Poaceae species: 50 Panicoideae, 35 new in this study, and the outgroup species. An alignment containing 72 complete plastomes, excluding one copy of the IR, was generated using Geneious Pro with the MAFFT v7.017 [38] plugin using the auto function for the algorithm and default settings. The alignment was then manually examined for any shared or unique rare genomic changes. Any gaps that were introduced in one or more sequences by the alignment were excluded from the matrix.

A model for the gap-free nucleotide alignment was selected using jModelTest version 2.1.3 [41, 42] on the gap-free nucleotide alignment, and the GTR + I + G model was selected under the Akaike information criterion [43]. Maximum parsimony (MP) [44], maximum likelihood (ML) [45], and Bayesian MC3 inference analyses (BI) [46] were performed on the data set. The MP analysis was performed with PAUP* version 4.0b10 [47]. The ML analysis used RAxML-HPC2 on XSEDE version 8.1.11 [48] at the CIPRES Science Gateway [49]. The number of bootstrap replicates was set to 1000, all model parameters were estimated during the analysis by default. The BI analysis used MrBayes on XSEDE version 3.2.3 [50] at the CIPRES Science Gateway [49]. MrBayes was set for two independent runs with four chains and twenty million generations each, with a default 25 % burn-in value. The substitution model was set to “invgamma” and “nst = 6” and the other parameters were set at defaults. Finally, SH tests [51] were used to test the best ML tree topology against seven constrained topologies as suggested by previous studies [14, 15, 17].

### Sanger sequencing verification of mtDNA inserts

A verification step was done to determine the mtDNA inserts in the plastomes of *P. dilatatum* and *P. fimbriatum*. Primers that were specific to only the mtDNA inserts were created (Additional file 4: Table S3). Each of these primers was used in combination with a plastome-specific primer in PCR experiments following the general methods of Dhingra and Folta [52] with modifications based on Leseberg and Duvall [53] and Morris and Duvall [20]. PCR products were electrophoretically separated for product number and length verification (Additional file 5: Figure S1). The products were cleaned using the Wizard SV PCR Clean-up System (Promega, Madison, Wisconsin, USA), and sent for automated Sanger sequencing at ACGT Inc. (Wheeling, Illinois, USA). The Geneious Pro pairwise align feature was used to align the Sanger and Illumina sequence data for verification of the inserts.

## Results

### Plastome assembly and feature analysis

Complete plastomes from 35 species of Panicoideae were annotated and submitted to Genbank. All plastomes were highly conserved and exhibited the gene content, gene order and the quadripartite structure that are typical of grasses. The length of the Panicoideae plastomes ranged from 134,520–141,182 bp with an average length of 139,687 bp. The number of reads in the new species files were on average 8,322,030, which assembled into an average of 4.89 contigs after *de novo* assembly. The complete plastomes were assembled with an average read depth of 114.76 (Additional file 2: Table S2).

The observed plastome length variation is primarily due to the *trnI-CAU****—****trnL-CAA* spacer. This region is located in the IR, and changes to its size are reflected twice in the overall plastome length. There are two different large inserts in *Paspalum fimbriatum* (2,878 bp) and *P. dilatatum* (963 bp) and one large deletion in *Panicum capillare* (~2,682 bp) making it the smallest plastome (134,520 bp). The longest plastome, *Saccharum officinarum* (141,097 bp), has no identifiable insert(s) to explain its unique length.

**Fig. 1:**
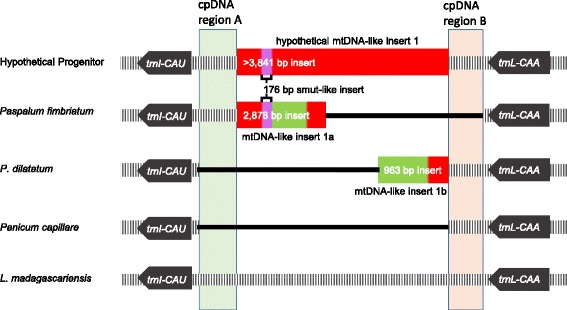
Plastome maps illustrating the mtDNA inserts in the *trnI* - *trnL* intergenic spacer in *Paspalum fimbriatum*, *P. dilatatum* and their probable progenitor, as well as *Panicum capillare*, which has a large deletion in this region, and *L. madagascariensis*, which illustrates a typical grass plastome. The colors correspond to the similarity of DNA determined by BLAST results: red is mitochondrial-like, purple is smut-like, and green is sequence that did not return high similarity scores with any banked sequences. The ptDNA regions A and B correspond to regions of DNA with high pairwise identity, and is present in most species of Panicoideae

The inserts in *P. fimbriatum* and *P. dilatatum* were queried for putative homology using BLASTn [40] and reads were mapped to both inserts to inspect continuity before, throughout, and after the putative mtDNA inserted sequence. In *P. fimbriatum*, the first 1,427 bp and the last 710 bp matched that to *Tripsacum dactyloides* mitochondrial DNA (mtDNA) at 98 % and 99 %, respectively. The internal 741 bp of the insert matched small sequences of non-grass DNA with a 176 bp read that resembles fungal *Melanopsichium pennsylvanicum* and *Ustilago maydis* rRNA at 68 % and 67 % identity, respectively. The read depth at the beginning of the insert in *P. fimbriatum* was 72 reads, 91 reads at the end of the insert, and an average of 117.8 reads for the entire insert compared to an average of 113 reads for the entire IR. The insert in *P. dilatatum* had multiple small inserts, collectively 346 bp, that matched to *Tripsacum dactyloides* mtDNA at 84 %. The rest of the sequence had smaller intermittent hits that resembled nuclear or mRNA of Poaceae (Fig. 1). The read depth at the beginning of the insert in *P. dilatatum* was 31 reads, 28 reads at the end of the insert, and an average of 23.6 reads for the entire insert compared to an average of 25.1 reads for the entire IR.

Based on the alignment, the gene *InfA* was noted as having two different start positions in Panicoideae. An upstream start was seen in 13 species: *Arundinella deppeana*, *Otachyrium versicolor*, *Steinchisma laxa*, *Plagiantha tenella*, *Coleataenia prionitis*, *Paspalum fimbriatum*, *Paspalum dilatatum*, *Paspalum glaziovii*, *Axonopus fissifolius*, *Setaria italica*, *Paspalidium geminatum*, *Lecomtella madagascariensis*, and *Thysanolaena latifolia*. The start codon occurs 18 bp further upstream compared to other Panicoideae due to a deletion event. With this deletion, there is also a base change at the beginning of the sequence, A**G**GA**G**A to A**T**GA**C**A, in the species with the smaller *InfA* gene. This changes the sequence to a start codon and keeps the rest of the coding sequence in frame. *Cenchrus americanus* also shows this same base change, but still has the longer *InfA* gene.

### Phylogenomic analyses

**Fig. 2:**
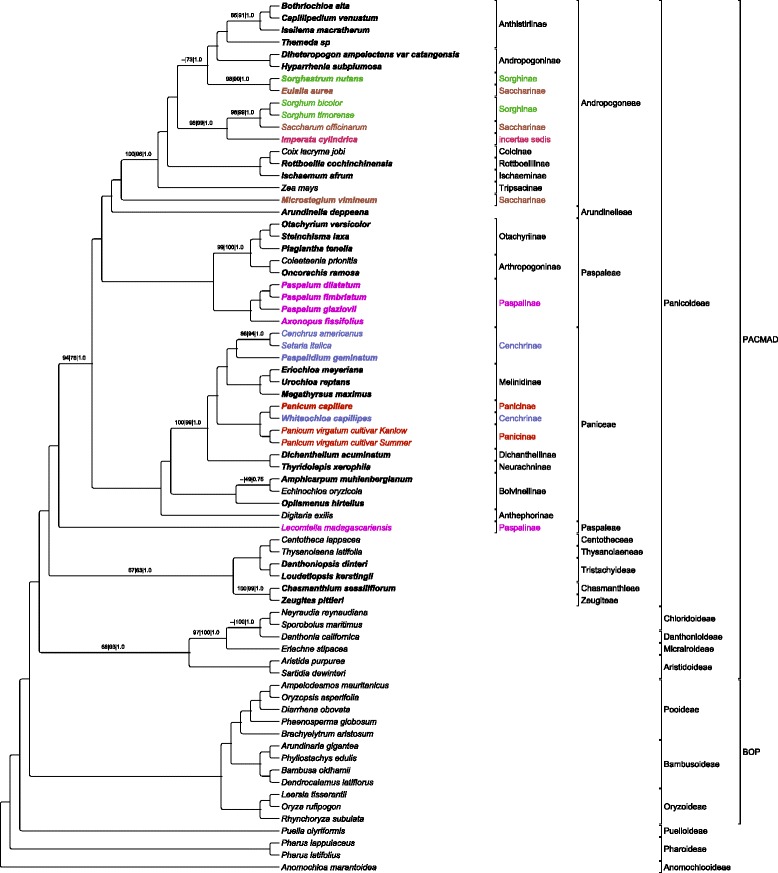
Cladogram of the 50 Panicoideae (species names in bold indicate that they are newly sequenced in this study) with outgroup species. All nodes are supported at bootstrap values (BV) of 100 and posterior probabilities (PP) of 1.0 except where noted (MPBV | MLBV | PP). Bootstrap analysis did not support branches marked “--”. Subtribal names with disagreements to monophyly are color coded. Tribes and higher taxonomic groupings are indicated

The alignment of the 72 species with one IR and gaps removed contained 12,757 parsimony informative characters. The MP analysis produced one most parsimonious tree with a consistency index (CI) excluding uninformative characters of 0.5032 and a retention index (RI) of 0.7608. The ML tree had -lnL = −417978.62 that was topologically identical to the consensus BI tree (Fig. 2). The MP tree differed from the ML tree and the consensus BI in three specific ways. The first difference was a polytomy including the clade of *Eulalia aurea* and *Sorghastrum nutans*, the clade of *Imperata cylindrica*, *Saccharum officinarum*, *Sorghum timorense* and *S. bicolor*, and the rest of the Andropogoninae and Anthistiriinae. The second difference was the topology among the three taxa: (*Amphicarpum muhlenbergianum*, (*Echinochloa oryzicola*, *Oplismenus hirtellus*)) in the MP tree and ((*A. muhlenbergianum*, *E. oryzicola*), *O. hirtellus*) in the ML tree. The final difference was the divergence pattern in the other PACMAD species, with the topology (Chloridoideae, (Danthonioideae, Micrairoideae)) in the MP tree, and (Micrairoideae, (Chloridoideae, Danthonioideae)) in the ML tree. In the topologies obtained from the three methods, Panicoideae were sister to the remainder of PACMAD species.

The phylogenomic analyses retrieved trees with six monophyletic groups corresponding to subtribes: Andropogoninae, Anthistiriinae, Arthropogoninae, Boivinellinae, Melinidinae, and Otachyriinae. Other areas of the final tree topologies indicated non-monophyly of five previously recognized subtribes. Two instances were found in Andropogoneae. The first is the placement of *Sorghastrum nutans* (Sorghinae) as sister to *Eulalia aurea* (Saccharinae). This clade was sister to a clade of Andropogoninae (two species) and Anthistiriinae (four species). The other Saccharinae, *Microstegium vimineum*, was sister to the remaining Andropogoneae. In Paniceae, *Whiteochloa capillipes* (Cenchrinae) was strongly supported (100|100|1.0) as the sister to *Panicum capillare*, and this clade was sister to *Panicum virgatum* (both Panicinae). The final non-monophyly was *Lecomtella madagascariensis* (Paspalinae*)* as sister to a clade of the four tribes: Andropogoneae, Paniceae, Paspaleae (in which the remaining Paspalinae are classified), and Arundinelleae. The five subtribes retrieved as non-monophyletic were then individually constrained as monophyletic as alternative topologies for the SH tests for comparison against the ML topology. Similarly, Aristidoideae was constrained as sister to the remaining PACMAD clade instead of Panicoideae testing previous alternative hypothesis for this subfamily [16]. Finally, all of the constrained groups were included in a single alternate tree. The SH tests rejected all alternative topologies compared to the ML tree topology (*p* < 0.05; Additional file 4: Table S4).

### Sanger sequencing verification of mtDNA insert

The amplifications produced one product of the expected size for each primer pair (Additional file 5: Figure S1). The reaction product for IRB 3 F/PdiR was 1109 | ~1100 bases (expected length | observed length), PdiF/IRCL 11R was 1170 | ~1200, IRB 10 F/PfiR was 1638 | ~1600, and PfiF/IRCL 11R was 1856 | ~1800. The sequence data for each species from the Sanger method matched the NGS sequence of the putative insert with 100 % nucleotide site identity verifying the boundaries between the mtDNA and the ptDNA for both species.

## Discussion

In this study, genome-skimming techniques were used to successfully generate 35 complete Panicoideae plastomes without plastid enrichment of the libraries. The three different library preps, TrueSeq, Nextera and NexteraXT, produced libraries that range from 1.1 - 27.7 million reads. Assembly methods of Wysocki et al. [33] produced on average 4.9 contigs per plastome (Additional file 2: Table S2). The inability of the software to create one large contig from the reads and contigs is mostly due to repetitive regions, which causes discontinuities in the de Bruijn graph and results in collapsing identical sequences [54]. Using quality trimmed reads to manually extend contigs until there was sufficient overlap between contigs resolved this problem.

### Plastome feature analysis

mtDNA to ptDNA transfers were originally thought to be extremely rare [55] or non-existent events [56, 57]. The first two documented cases of mtDNA to ptDNA transfer were discovered in the eudicots *Daucus carota* [58] and *Asclepias syriaca* [59]. The first instance of mtDNA to ptDNA transfer in monocots was found in two genera of the subtribe Parianinae of the Olyreae (herbaceous bamboos *Eremitis* sp. and *Pariana radiciflora*) by Wysocki et al. [25] and was subsequently confirmed in two other Parianinae species [60]. Another instance was found in *Triticum monococcum* [23]. The sequence in *Paspalum dilatatum* and *P. fimbriatum* plastomes with high sequence similarity to mtDNA, which were sequenced in this study, provide further evidence of mtDNA to ptDNA transfer. Neither extracts nor libraries were enriched for any specific type of DNA, and the read depths of each insert are similar to the read depth of the IR region in which it is found. On average there are around 50 chloroplasts per mesophyll cell, but hundreds to thousands of mitochondria per average cell [61]. Therefore, if these inserts were created by assembly error, and the sequence is actually found solely in the mitochondrial genome, we would expect much greater read depth in the putative inserts than the average read depth of the plastid IR. This magnitude of difference would be reflective of the greater number of mitochondria per average cell. It is also important to note that flanking regions in the assembly showed no sign of mismatching, misassembly, or coverage interruption.

The mtDNA inserts in the plastome of *P. fimbriatum* were queried using BLASTn and matched to the *Tripsacum dactyloides* cultivar Pete (Andropogoneae) mitochondrion (GenBank accession NC_008362) at 98 % identity. Note that there are relatively few complete mitogenomes sequenced from Poaceae. The upstream part of the insert had a 98 % nucleotide identity to the mitochondrial *trnM-a-*1 ***—****trnY-*1 intergenic region in *T. dactyloides* and further downstream in a region that repeats these same genes, *trnM-a-*2 ***—****trnY-*2 intergenic region. The downstream mtDNA insert had a 99 % nucleotide identity to the nad4L ***—****trnF-cp* intergenic region. The distance between these regions in the plastome is 741 bp, compared to *T. dactyloides* where it is over 187,000 bp from the *trnM-a-*1 ***—****trnY-*1 intergenic region or over 122,000 bp from the *trnM-a-*2 ***—****trnY-*2 intergenic region. This means that the mtDNA to ptDNA transfer was either: 1) two separate rare transfer events in congeners, or 2) that the gene and intergenic region order in the mtDNA donor to *P. fimbriatum* differs greatly from that of *T. dactyloides*. Difference in gene order is probable since differences in mitochondrial gene order between genera and even congeneric species is reported [62–65].

The mtDNA insert in *P. fimbriatum* also contained a 176 bp sequence that returned a BLAST query with the top results being *Melanopsichium pennsylvanicum* (HG529787) and *Ustilago maydis* (NC_008368), smut fungi (Ustilaginaceae) that usually infect Poaceae [66]. There are two possible explanations for the smut-like insert. The first is that two insertions occurred in the plastome in which a smut-like sequence was inserted into an already existing mitochondrial-like insert. The other scenario is one insertion of a mitochondrial sequence into the plastome that has the smut-like sequence already acquired by the mitochondrion. The latter seems more probable since plant mitochondria will actively import foreign DNA on a regular basis [67], while uptake of foreign DNA into the plastome is an extremely rare event [55]. The second scenario is also preferable due to the smut’s placement between two mtDNA sequences, and the overall unlikeliness of two insertions, one nested within the other, occurring in the plastome.

The mtDNA insert in *P. dilatatum* is not as readily explained. The BLAST results from *P. dilatatum* also correspond with *T. dactyloides* at 84 % identity, but the results are small and fragmented sequences: 179 bp, 98 bp and 69 bp. None of the mtDNA sequences in the *P. dilatatum* insert are similar to the mtDNA sequences in the *P. fimbriatum* insert, and the insert in *P. dilatatum* is noticeably shorter, a difference of 1,915 bp.

One hypothesis for the presence of the inserted region is that since *P. fimbriatum* and *P. dilatatum* are two congeners with an extremely rare mtDNA insert, the event could have happened once in their immediate progenitor. But to explain the fact that the mtDNA inserts have little sequence homology, subsequent independent mutations degrading this mtDNA sequence separately in each species would have to be postulated. A second hypothesis is that *P. fimbriatum* and *P. dilatatum* had parallel, independent mtDNA insertions. However, the rarity of these events [55] reduces the probability of this scenario. Other hypothetical scenarios are also possible, but even more complex.

The *trnI****—****trnL* intergenic spacer region, the region in which the mtDNA insert occurs, appears to be a hotspot region for deletions in grasses. Most non-grass plants have a functional *ycf2* gene in the *trnI****—****trnL* intergenic spacer region. With the elimination of the *ycf2* gene in grasses, this could lead to differential degradation as hypothesized by Maier et al. [68]. All outgroup grass species in this study have a deletion of at least ~1,141 bp within this region, while most Panicoideae have a ~392 bp deletion except for *Digitaria exilis*. In the Panicoideae, there are multiple unique deletions in this region such as a ~1,018 bp deletion in *Oplismenus hirtellus*, a ~371 bp deletion shared by *Setaria italica* and *Paspalidium geminatum*, and a ~349 bp deletion in *Digitaria exilis*. A larger deletion, ~3,611 bp, is found here in *Panicum capillare*. Part of the deletion in *P. capillare* encompasses ptDNA region A, a region of ptDNA that is shared by most Panicoideae species (Fig. 1), and the deletion of this region is also a shared in *P. dilatatum*. A similar deletion is seen in *P. fimbriatum*, where there is a deletion in the ptDNA region B (Fig. 1), another region shared by most species of Panicoideae. No flanking repeats or other sequences suggested a specific mutational mechanism. However, the mechanism that is causing the deletions is not specific to one lineage, but is occurring independently in different lineages thus indicating that deletions are common in this region.

Based on the data presented, this study suggests that the size of the putative mtDNA insert in the plastome would be at least 3,841 bp, the combined lengths of the two inserts in *P. dilatatum* and *P. fimbriatum* (Fig. 1). Then the same type of elimination/degradation that is seen in some panicoids for this region, also occurred in the ancestor of *P. fimbriatum* and *P. dilatatum*. This could also obscure the original mtDNA sequence, thus making it more difficult to recognize. This may explain the large portions of sequence, 565 bp in *P. fimbriatum* and 617 bp in *P. dilatatum*, that do not return BLAST results with high identities to banked sequences over 30 bp in size. Thus, due to different molecular histories with non-uniform degradation of the original mitochondrial insertion, the single insertion appears to be two different insertions in congeners.

The other notable molecular event in the panicoid plastomes was the shortening of the *infA* gene. This event, while homoplasious, identifies another hotspot for mutation in the grass plastome. The original sequence of T|ATG|ACAGAAAAAAAAAATAGGAGAGAAAAAAAAAA, where the start codon in frame is bracketed with pipes, exhibits two SNPs, seen in *Cenchrus americanus* that change it to: T|ATG|ACAGAAAAAAAAAAT|A**T**G|A**C**AGAAAAAAAAAA. These two SNPs convert it to a perfect 18 bp repeat, which is then sometimes lost, presumably by slipped strand mispairing [69]. The sequence is deleted in *Setaria italica*, *Paspalidium geminatum*, *Arundinella deppeana*, *Lecomtella madagascariensis*, and *Thysanolaena latifolia* as well as in most Paspaleae except for *Oncorachis ramosa*.

### Phylogenomic analyses

The phylogeny that was estimated (Fig. 2) is in partial agreement with previous studies [14–16]. Following the taxonomy in most of these studies, we retrieved two of the major tribes, Andropogoneae and Paniceae, as monophyletic, as well as the smaller tribe Tristachyideae. The general order of divergence is congruent with GPWG II [16] and Soreng et al. [15]. The five smaller tribes, Centotheceae, Chasmanthieae, Thysanolaeneae, Tristachyideae, and Zeugiteae, diverge as one clade with subsequent within-group divergences, which is not consistent with Morrone et al. [14]. This is followed in general by the serial divergence of Paniceae, Paspaleae, Arundinelleae and Andropogoneae. Taxa that do not demonstrate monophyly or show relationships that are strongly incongruent with previous studies [14–16] are noted below.

### Saccharinae and Sorghinae

Within the Andropogoneae, the sister group relationship between *Sorghastrum nutans* and *Eulalia aurea* is highly supported (98|90|1.00), but their placement in a clade with Anthistiriinae and Andropogoninae has much lower support (−−|73|1.00) (Fig. 2). Both alternative trees that forced the monophyly of Saccharinae and Sorghinae were rejected as plausible alternative hypotheses by SH tests (Fig. 2). Future sampling with better balancing of ingroup Sorghinae and Saccharinae with outgroup Andropogoneae might better address questions of subtribal monophylies. The other member of Saccharinae that contradicts the monophyly of the subtribe is *Microstegium vimineum. M. vimineum* has been under scrutiny due to suspected non-monophyly of this genus and uncertain, weakly supported placement [70]. This study produced maximum support, from all three analyses, for the placement of *M. vimineum* as sister to the rest of the Andropogoneae. While this placement of *M. vimineum* is congruent with previous results for this species [70], whole plastome sequences of *M. nudum* and other species of *Microstegium* will be required to demonstrate the full extent of non-monophyly of this genus.

### Imperata cylindrica

The genus *Imperata* is classified as *incertae sedis* within Andropogoneae by Soreng et al. [15]. The placement of *I. cylindrica* in this study has strong support (98|99|1.0) as sister to a clade of *Saccharum officinarum* and two species of *Sorghum*. This is in disagreement with previous studies of Panicoideae that suggest a sister relationship for *I. cylindrica* with the subtribe Germainiinae [16, 71]. This uncertain placement stems from the reorganization of the Saccharinae and Germainiinae, which left four genera, *Eriochrysis*, *Imperata*, *Pogonatherum,* and *Tripidium,* as *incertae sedis* [15]. More extensive sampling of complete plastomes is needed before recommendations can be made for the confident placement of *I. cylindrica* or any of these other genera.

### Panicinae and Cenchrinae

Within the Paniceae, another incongruence with other studies is the placement of *Whiteochloa capillipes* (Cenchrinae) within the subtribe Panicinae. Previous studies have placed *W. capillipes* within Cenchrinae, but with low branch support or polytomies in the subtending nodes [14, 16]. All three analyses produce maximum support for the placement of *W. capillipes* within Panicinae in this study. The alternative tree that constrained the monophyly of Cenchrinae was rejected as plausible alternative hypotheses by SH tests (Fig. 2). While we are not making a recommendation for reclassification of *W. capillipes*, with only six species of *Whiteochloa* [72] an analysis of a more taxonomically dense matrix including all species from this genus could be performed using complete plastomes to determine the phylogenomic placement of this genus within Paniceae.

### Lecomtella madagascariensis

The final subtribal discrepancy is the positioning of *Lecomtella madagascariensis* in Paspaleae. There is high contrast between the placement of *L. madagascariensis* in this study, which is sister to a clade of the four tribes Paniceae, Paspaleae, Arundinelleae and Andropogoneae, and two previous studies based on considerably less plastome sequence that place it in a weakly supported Paspalinae [14, 15]. Our phylogenomic hypothesis, which is supported by SH tests and moderate support values (94|75|1.00), is more congruent with a study specifically investigating the placement of *L. madagascariensis* [28]. Here, the full plastome history that occurred during the approximately 20 million years of isolated evolution in Madagascar [28] informs our hypothesis. Based on this study and the recent study by Besnard et al. [28] we strongly support the adoption of the tribe Lecomtelleae.

### PACMAD clade

The dominant hypothesis for the deep divergences in this clade was the Aristidoideae in a weakly supported position as the sister to the other five subfamilies [16]. An alternate hypothesis was first proposed by Cotton et al. [17] suggesting that Panicoideae occupied a sister position to the remaining subfamilies and this result was retrieved despite the composition of outgroups. Further support for this alternate hypothesis was found by Duvall et al. [31] when 14 chloridoid species were included. Here we show that increasing panicoid sampling similarly supports the alternative hypothesis. Early divergence in PACMAD grasses has important implications for the origin and evolution of the group, but currently no data set has been reported to definitively reject either the original or alternative hypothesis.

## Conclusion

In conclusion 35 new Panicoideae species were sequenced via NGS technologies. An investigation of rare genomic changes was conducted and two instances of mtDNA inserts in the plastome were added to a growing list of probable mtDNA to ptDNA transfer events among grasses. A phylogeny was retrieved based on 50 panicoids, which strengthened previous results in some cases, but also identified some incongruencies that require more taxonomic sampling for certain genera. Future Panicoideae studies could also benefit from having at least one representative from each tribe, as this would help with the placement of *incertae sedis* species as well as uncertain tribal relationships in the subfamily. We also endorse the adoption of the tribe Lecomtelleae, based on the new robust phylogeny from this paper as well as a previous study [28]. Finally this study demonstrates a new level of phylogenomic sampling, 72 species, at which complete plastome studies can be conducted, while still maintaining high resolution and support.

## Abbreviations

ACRE, anchored conserved region extension; bp, base pair; BI, bayesian Inference; BLAST, Basic Local Alignment Search Tool; BOP, Bambusoideae Oryzoideae Pooideae; BV, bootstrap value; CI, consistency index; CIPRES, cyber infrastructure for phylogenetic research; Contigs, contiguous sequences; GPWG (II), grass phylogeny working group (II); GTR + G + I, general time reversible plus gamma distribution plus proportion of invariant sites; IR, inverted repeat; MAFFT, multiple alignment using fast fourier transform; ML, maximum likelihood; MLBV, maximum likelihood bootstrap value; MP, maximum parsimony; MPBV, maximum parsimony bootstrap value; mtDNA, mitochondrial DNA; NCBI, National Center for Biotechnology Information; NGS, next generation sequencing; PAUP*, Phylogenetic Analysis Using Parsimony * and other methods; PACMAD, Panicoideae Arundinoideae Chloridoideae Micrairoideae Arundinoideae Danthonioideae; PP, Posterior probability; ptDNA, plastid DNA; RI, retention index; SH test, Shimodaira Hasegawa test; SNP, single nucleotide polymorphism; SSC, short single copy

## Additional files

Additional file 1: Table S1. Plastomes analyzed in this study with NCBI accession numbers and lengths of subregions and complete Panicoideae plastomes. (DOCX 20 kb) Additional file 2: Table S2. Next generation sequencing details for newly assembled Panicoideae plastomes from this study. (DOCX 15 kb) Additional file 3: Table S3. The specific mtDNA primers that were created to verify the inserts in *P. dilatatum* (Pdi) and *P. fimbriatum* (Pfi). (DOCX 12 kb) Additional file 4: Table S4. The results of the SH tests. (DOCX 12 kb) Additional file 5: Figure S1. Gel image for PCR experiment conducted to verify mitochondrial insertions. Primer pairs are listed and informative fragment lengths of standard fragments are indicated in bases. Pdi = P. dilatatum; Pfi = P. fimbriatum. (PDF 210 kb)
