# Supplementary material for: Evolutionary relationships in Panicoid grasses based on plastome phylogenomics (Panicoideae; Poaceae)
Source: BMC Plant Biol. 2016 Jun 18;16:140. doi: 10.1186/s12870-016-0823-3 (PMC4912804; doi:10.1186/s12870-016-0823-3)
Supplement: Additional file 1: Table S1. — Plastomes analyzed in this study with NCBI accession numbers and lengths of subregions and complete Panicoideae plastomes. (DOCX 20 kb) [file 12870_2016_823_MOESM1_ESM.docx]

Supplemental 1: Plastomes analyzed in this study with NCBI accession numbers and lengths of subregions and complete Panicoideae plastomes.

| Subfamily | Tribe | Taxon | Voucher | Accession | Total Length | LSC^a^ | SSC ^b^ | IR ^c^ |
| --- | --- | --- | --- | --- | --- | --- | --- | --- |
| Panicoideae | Andropogoneae | ***Bothriochloa alta*** | Duvall s. n. (DEK) | KU291492 | 137,645 | 79,532 | 12,543 | 22,785 |
|  |  | ***Capillipedium venustum*** | PI 11713 | KU291493 | 138,257 | 80,131 | 12,496 | 22,815 |
|  |  | ***Diheteropogon amplectens* var. *catangensis*** | PI 12585 | KU291497 | 139,700 | 81,479 | 12,611 | 22,805 |
|  |  | ***Eulalia aurea*** | PI 12153 | KU291499 | 140,220 | 82,112 | 12,546 | 22,781 |
|  |  | ***Hyparrhenia subplumosa*** | PI 12665 | KU291500 | 139,138 | 81,229 | 12,603 | 22,653 |
|  |  | ***Imperata cylindrica*** | Burke 21 (DEK) | KU291466 | 140,744 | 82,649 | 12,541 | 22,777 |
|  |  | ***Ischaemum afrum*** | PI 364924 | KU291467 | 141,097 | 82,984 | 12,567 | 22,773 |
|  |  | ***Iseilema macratherum*** | PI 257760 | KU291468 | 139,643 | 81,724 | 12,567 | 22,676 |
|  |  | ***Microstegium vimineum*** | PI 659331 | KU291471 | 140,460 | 82,382 | 12,542 | 22,768 |
|  |  | ***Rottboellia cochinchinensis*** | Clark et al. 1698 (ISC) | KU291481 | 140,336 | 82,298 | 12,550 | 22,744 |
|  |  | ***Sorghastrum nutans*** | Wysocki s.n. (DEK) | KU291482 | 141,061 | 82,907 | 12,544 | 22,805 |
|  |  | ***Themeda* sp.** | Saarela 1833 (CAN) | KU291484 | 138,842 | 80,730 | 12,546 | 22,783 |
|  |  | *Saccharum officinarum* | N/A | NC_006084 | 141,182 | 83,048 | 12,544 | 22,795 |
|  |  | *Zea mays* | N/A | NC_001666 | 140,384 | 82,352 | 12,536 | 22,748 |
|  |  | *Coix lacryma-jobi* | Duvall s.n. (DEK) | NC_013273 | 140,745 | 82,792 | 12,523 | 22,715 |
|  |  | *Sorghum bicolor* | N/A | NC_008602 | 140,754 | 83,733 | 12,503 | 22,259 |
|  |  | *Sorghum timorense* | N/A | NC_023800 | 140,629 | 83,471 | 12,480 | 22,339 |
|  | Arundinelleae | ***Arundinella deppeana*** | Clark et al. 1680 (XAL) | KU291490 | 140,804 | 82,619 | 12,549 | 22,818 |
|  | Chasmanthieae | ***Chasmanthium sessiliflorum*** | Sánchez-Ken s.n. (ISC) | KU291494 | 140,892 | 82,772 | 12,624 | 22,748 |
|  | Centotheceae | *Centotheca lappacea* | N/A | NC_025229 | 139,464 | 81,451 | 12,553 | 22,730 |
|  | Paniceae | ***Amphicarpum muhlenbergianum*** | Clark et al. 1695 (ISC) | KU291489 | 140,810 | 82,787 | 12,641 | 22,691 |
|  |  | ***Dichanthelium acuminatum*** | Saarela 666 (CAN) | KU291496 | 140,122 | 82,065 | 12,617 | 22,720 |
|  |  | ***Eriochloa meyeriana*** | Duvall s.n. (DEK) | KU291498 | 139,890 | 81,856 | 12,568 | 22,733 |
|  |  | ***Megathyrsus maximus*** | PI 12181 | KU291470 | 138,994 | 81,044 | 12,524 | 22,713 |
|  |  | ***Oplismenus hirtellus*** | Clark & Lewis 1644 (ISC) | KU291473 | 137,424 | 81,421 | 12,575 | 21,714 |
|  |  | ***Panicum capillare*** | Saarela 769 (CAN) | KU291475 | 134,520 | 81,858 | 12,576 | 20,043 |
|  |  | ***Paspalidium geminatum*** | Giussani 313 (SI) | KU291476 | 139,142 | 82,041 | 12,437 | 22,332 |
|  |  | ***Thyridolepis xerophila*** | Saarela 1643 (CAN) | KU291485 | 140,644 | 82,641 | 12,599 | 22,702 |
|  |  | *Cenchrus americanus* | N/A | NC_024171 | 140,718 | 91,260 | 12,576 | 18,441 |
|  |  | *Setaria italica* | N/A | KJ001642 | 138,833 | 81,916 | 12,529 | 22,194 |
|  |  | *Panicum virgatum* cultivar Summer | N/A | HQ822121 | 139,619 | 81,659 | 12,562 | 22,699 |
|  |  | *Panicum virgatum* cultivar Kanlow | N/A | HQ731441 | 139,677 | 81,729 | 12,538 | 22,705 |
|  |  | *Echinochloa oryzicola* | N/A | NC_024643 | 139,891 | 82,108 | 12,515 | 22,634 |
|  |  | *Digitaria exilis* | N/A | NC_024176 | 140,908 | 91,883 | 12,637 | 18,194 |
|  |  | ***Urochloa reptans*** | Morden 1221 (HAW) | KU291486 | 140,171 | 82,157 | 12,590 | 22,712 |
|  |  | ***Whiteochloa capillipes*** | Duvall s.n. (DEK) | KU291487 | 139,177 | 81,199 | 12,536 | 22,721 |
|  | Paspaleae | ***Axonopus fissifolius*** | Clark et al. 1703 (ISC) | KU291491 | 139,250 | 81,144 | 12,588 | 22,759 |
|  |  | ***Otachyrium versicolor*** | Zuloaga 7027 (SI) | KU291474 | 140,081 | 81,908 | 12,721 | 22,726 |
|  |  | ***Paspalum dilatatum*** | Peterson 19673 (CAN) | KU291477 | 135,950 | 81,293 | 12,613 | 21,022 |
|  |  | ***Paspalum fimbriatum*** | Morrone 3651 (SI) | KU291478 | 140,804 | 80,782 | 12,600 | 23,711 |
|  |  | ***Paspalum glaziovii*** | Filgueiras 3482 (SI) | KU291479 | 139,255 | 81,115 | 12,602 | 22,769 |
|  |  | *Coleataenia prionitis* | N/A | NC_025231 | 139,897 | 81,886 | 12,551 | 22,730 |
|  |  | ***Plagiantha tenella*** | Zuloaga 6953 (SI) | KU291480 | 139,722 | 81,714 | 12,622 | 22,693 |
|  |  | ***Steinchisma laxa*** | Zuloaga 7416 (SI) | KU291483 | 139,902 | 81,735 | 12,653 | 22,757 |
|  |  | ***Oncorachis ramosa*** | Zuloaga 6960 (SI) | KU291472 | 139,871 | 81,778 | 12,663 | 22,715 |
|  | Thysanolaeneae | *Thysanolaena latifolia* | N/A | NC_025238 | 140,097 | 82,173 | 12,466 | 22,729 |
|  | Tristachyideae | ***Danthoniopsis dinteri*** | Duvall s.n. (DEK) | KU291495 | 139,169 | 81,054 | 12,617 | 22,749 |
|  |  | ***Loudetiopsis kerstingii*** | PI 12679 | KU291469 | 138,377 | 80,332 | 12,599 | 22,723 |
|  | Zeugiteae | ***Zeugites pittieri*** | Clark et al. 1171 (ISC) | KU291488 | 139,738 | 81,612 | 12,632 | 22,747 |
| Anomochlooideae | *Anomochloa marantoidea* NC_014062 | | | | | | | |
| Aristidoideae | *Aristida purpurea* NC_025228; *Sartidia dewinteri* NC_027147 | | | | | | | |
| Bambusoideae | *Arundinaria gigantea* NC_020341; *Bambusa oldhamii* NC_012927; *Dendrocalamus latiflorus* NC_013088; *Phyllostachys edulis* NC_015817 | | | | | | | |
| Chloridoideae | *Neyraudia reynaudiana* NC_024262; *Sporobolus maritimus* NC_027650 | | | | | | | |
| Danthonioideae | *Danthonia californica* NC_025232 | | | | | | | |
| Micrairoideae | *Eriachne stipacea* NC_025234 | | | | | | | |
| Oryzoideae | *Leersia tisserantii* NC_016677; *Oryza rufipogon* NC_022668; *Rhynchoryza subulata* NC_016718 | | | | | | | |
| Pharoideae | *Pharus lappulaceus* NC_023245; *Pharus latifolius* NC_021372 | | | | | | | |
| Pooideae | *Ampelodesmos mauritanicus* NC_027466; *Brachyelytrum aristosum* NC_027470; *Diarrhena obovata* KM974739; *Oryzopsis asperifolia* NC_027479; *Phaenosperma globosum* NC_027480 | | | | | | | |
| Puelioideae | *Puelia olyriformis* NC_023449 | | | | | | | |

^a^Large Single Copy Region.

^b^Short Single Copy Region.

^c^Inverted Repeat Region.
