# Supplementary material for: Evolutionary relationships in Panicoid grasses based on plastome phylogenomics (Panicoideae; Poaceae)
Source: BMC Plant Biol. 2016 Jun 18;16:140. doi: 10.1186/s12870-016-0823-3 (PMC4912804; doi:10.1186/s12870-016-0823-3)
Supplement: Additional file 2: Table S2. — Next generation sequencing details for newly assembled Panicoideae plastomes from this study. (DOCX 15 kb) [file 12870_2016_823_MOESM2_ESM.docx]

Supplemental 2: Next generation sequencing details for newly assembled Panicoideae plastomes from this study.

| Tribe | Taxon | Number of Reads | Mean coverage | Number of scaffolded contigs | Library preparation method | *de novo* Assembler |
| --- | --- | --- | --- | --- | --- | --- |
| Andropogoneae | ***Bothriochloa alta*** | 3,952,511 | 54 | 3 | Nextera | Iterative Velvet |
|  | ***Capillipedium venustum*** | 3,985,354 | 167.3 | 6 | Nextera XT | SPAdes |
|  | ***Diheteropogon amplectens* var. *catangensis*** | 4,578,653 | 101.6 | 8 | Nextera XT | SPAdes |
|  | ***Eulalia aurea*** | 2,845,258 | 99.7 | 2 | Nextera | Iterative Velvet |
|  | ***Hyparrhenia subplumosa*** | 4,510,846 | 105.9 | 10 | Nextera XT | SPAdes |
|  | ***Imperata cylindrica*** | 7,814,541 | 92.9 | 6 | Nextera | Iterative Velvet |
|  | ***Ischaemum afrum*** | 6,565,246 | 280.6 | 5 | Nextera | Iterative Velvet |
|  | ***Iseilema macratherum*** | 4,306,783 | 59.6 | 6 | Nextera | Iterative Velvet |
|  | ***Microstegium vimineum*** | 4,072,254 | 73.7 | 4 | Nextera | Iterative Velvet |
|  | ***Rottboellia cochinchinensis*** | 6,400,738 | 116.7 | 4 | Nextera | Iterative Velvet |
|  | ***Sorghastrum nutans*** | 8,716,655 | 115.5 | 2 | Nextera | Iterative Velvet |
|  | ***Themeda* sp*.*** | 9,482,109 | 106.7 | 4 | TruSeq | Iterative Velvet |
| Arundinelleae | ***Arundinella deppeana*** | 10,743,205 | 132.4 | 3 | Nextera | Iterative Velvet |
| Chasmanthieae | ***Chasmanthium sessiliflorum*** | 12,013,761 | 51.7 | 4 | Nextera | Iterative Velvet |
| Paniceae | ***Amphicarpum muhlenbergianum*** | 5,621,232 | 60.4 | 5 | Nextera | Iterative Velvet |
|  | ***Dichanthelium acuminatum*** | 14,887,340 | 131.1 | 4 | Nextera | Iterative Velvet |
|  | ***Eriochloa meyeriana*** | 2,066,815 | 24.1 | 4 | Nextera XT | SPAdes |
|  | ***Megathyrsus maximus*** | 3,640,935 | 149.4 | 6 | Nextera XT | SPAdes |
|  | ***Oplismenus hirtellus*** | 7,746,667 | 108.5 | 4 | Nextera | Iterative Velvet |
|  | ***Panicum capillare*** | 3,463,258 | 96.8 | 5 | Nextera | Iterative Velvet |
|  | ***Paspalidium geminatum*** | 8,249,096 | 71.4 | 4 | Nextera | Iterative Velvet |
|  | ***Thyridolepis xerophila*** | 16,576,393 | 188.5 | 2 | Nextera | Iterative Velvet |
|  | ***Urochloa reptans*** | 15,661,425 | 189.5 | 5 | Nextera | Iterative Velvet |
|  | ***Whiteochloa capilipes*** | 1,835,709 | 57.1 | 5 | Nextera XT | SPAdes |
| Paspaleae | ***Axonopus fissifolius*** | 26,882,182 | 296.2 | 4 | Nextera | Iterative Velvet |
|  | ***Otachyrium versicolor*** | 13,379,401 | 96.7 | 3 | TruSeq | Iterative Velvet |
|  | ***Paspalum dilatatum*** | 1,169,117 | 18.9 | 4 | TruSeq | Iterative Velvet |
|  | ***Paspalum fimbriatum*** | 8,321,619 | 52.7 | 4 | TruSeq | Iterative Velvet |
|  | ***Paspalum glaziovii*** | 27,770,279 | 80.3 | 7 | Nextera | Iterative Velvet |
|  | ***Plagiantha tenella*** | 4,455,738 | 44.4 | 2 | TruSeq | Iterative Velvet |
|  | ***Steinchisma laxa*** | 4,580,559 | 32.1 | 3 | Nextera | Iterative Velvet |
|  | ***Oncorachis ramosa*** | 6,338,398 | 28.6 | 7 | Nextera | Iterative Velvet |
| Tristachyideae | ***Danthoniopsis dinteri*** | 9,253,812 | 409.6 | 7 | Nextera | Iterative Velvet |
|  | ***Loudetiopsis kerstingii*** | 1,754,749 | 43.7 | 11 | Nextera XT | SPAdes |
| Zeugiteae | ***Zeugites pittieri*** | 17,628,413 | 278.2 | 8 | TruSeq | Iterative Velvet |
