# Supplementary material for: Evolutionary relationships in Panicoid grasses based on plastome phylogenomics (Panicoideae; Poaceae)
Source: BMC Plant Biol. 2016 Jun 18;16:140. doi: 10.1186/s12870-016-0823-3 (PMC4912804; doi:10.1186/s12870-016-0823-3)
Supplement: Additional file 3: Table S3. — The specific mtDNA primers that were created to verify the inserts in P. dilatatum (Pdi) and P. fimbriatum (Pfi). (DOCX 12 kb) [file 12870_2016_823_MOESM3_ESM.docx]

Supplemental 3: The specific mtDNA primers that were created to verify the inserts in *P. dilatatum* (Pdi) and *P. fimbriatum* (Pfi).

| Mito_Pdi_F | GGTCAAATGGACGACACCTGAAGGGG |
| --- | --- |
| Mito_Pdi_R | GGGTGGTTTTCGGTATGCTTCAGCG |
| Mito_Pfi_F | GAGTCTCCTCCCCTCATTTCGCTGC |
| Mito_Pfi_R | CCCCCTTATTTAGCTTACGCCCTTG |
