# Supplementary material for: Evolutionary relationships in Panicoid grasses based on plastome phylogenomics (Panicoideae; Poaceae)
Source: BMC Plant Biol. 2016 Jun 18;16:140. doi: 10.1186/s12870-016-0823-3 (PMC4912804; doi:10.1186/s12870-016-0823-3)
Supplement: Additional file 4: Table S4. — The results of the SH tests. (DOCX 12 kb) [file 12870_2016_823_MOESM4_ESM.docx]

Supplemental 5: The results of the SH tests.

| Constraints Applied | -ln L | Diff -ln L | P |
| --- | --- | --- | --- |
| Unconstrained (ML Tree) | 436593.90855 | (Best) |  |
| Cenchrinae, Panicinae, Paspalinae, Saccharinae, and Sorghinae Monophyletic | 440619.61968 | 4025.71113 | 0.000* |
| Cenchrinae Monophyletic | 438450.03649 | 1856.12794 | 0.000* |
| Panicinae Monophyletic | 436667.91684 | 74.00830 | 0.000* |
| Paspalinae Monophyletic | 438590.08577 | 1996.17722 | 0.000* |
| Saccharinae Monophyletic | 437033.74197 | 439.83342 | 0.000* |
| Sorghinae Monophyletic | 436751.71297 | 157.80443 | 0.000* |
| Aristidoideae sister to remaining PACMAD clade | 436635.76853 | 41.85998 | 0.032* |

*P<0.05
