# Supplementary material for: Evolutionary relationships in Panicoid grasses based on plastome phylogenomics (Panicoideae; Poaceae)
Source: BMC Plant Biol. 2016 Jun 18;16:140. doi: 10.1186/s12870-016-0823-3 (PMC4912804; doi:10.1186/s12870-016-0823-3)

Figure S1: Gel image for PCR experiment conducted to verify mitochondrial insertions. Primer pairs are listed and informative fragment lengths of standard fragments are indicated in bases. Pdi = *P. dilatatum;* Pfi = *P. fimbriatum.*

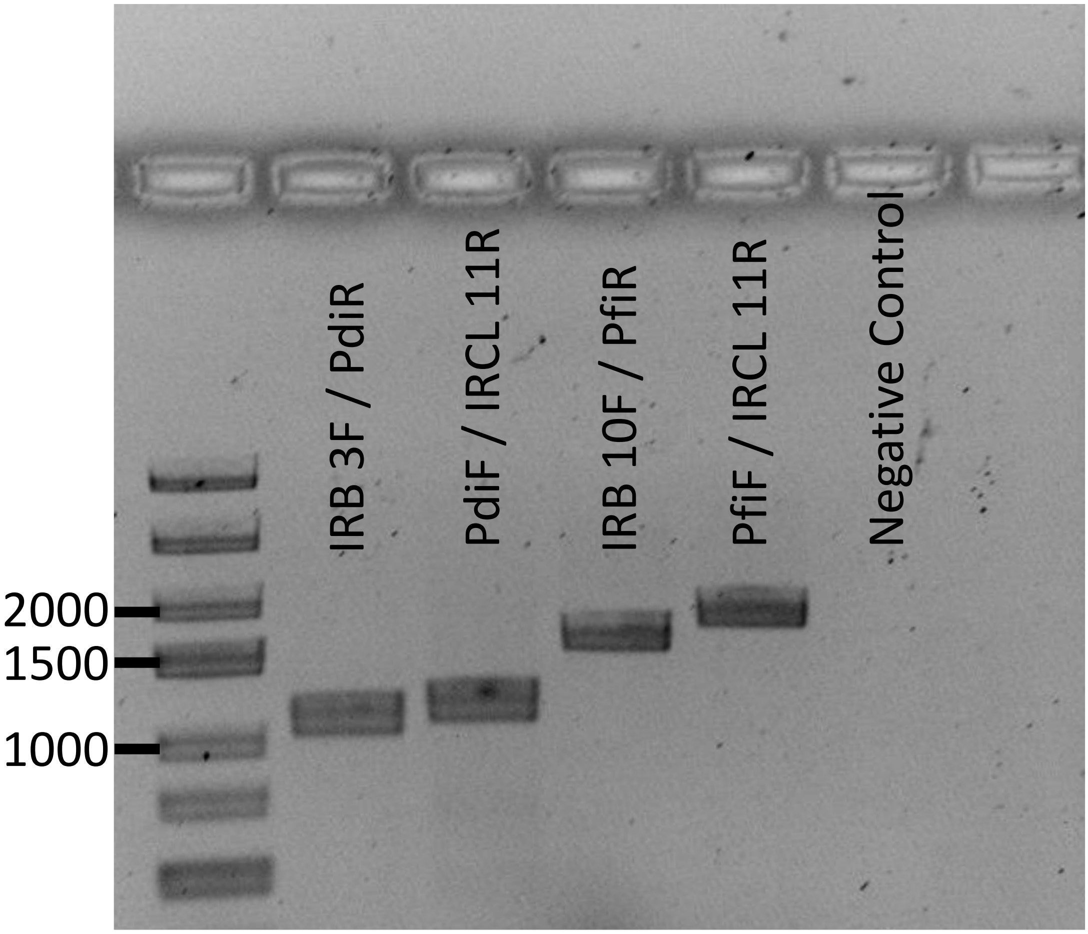

Supplement: Additional file 5: Figure S1. — Gel image for PCR experiment conducted to verify mitochondrial insertions. Primer pairs are listed and informative fragment lengths of standard fragments are indicated in bases. Pdi = P. dilatatum; Pfi = P. fimbriatum. (PDF 210 kb) [file 12870_2016_823_MOESM5_ESM.pdf]
